# Supplementary material for: Content-rich biological network constructed by mining PubMed abstracts
Source: BMC Bioinformatics. 2004 Oct 8;5:147. doi: 10.1186/1471-2105-5-147 (PMC528731; doi:10.1186/1471-2105-5-147)
Supplement: Additional File 2 — The original results of the above study (non-essential files are deleted to keep the file size under the limit set by BMC bioinformatics). [file 1471-2105-5-147-S2.bz2 › chilibotAdditionalFile2/dip05/405ID10681548E10065/html/IL6_OSM.html]

 


 **IL6** and **OSM** 
  
Found 446 abstracts in PubMed, retrieved 05.  
 

 What does Google say? 
 PDF only 
| .edu only 

---

**Interactive relationship** (e.g. stimulation, inhibition, etc)

**Neutral relationship**- Oncostatin M  [ **OSM** ]   **OSM**  is a multifucntinal cytokine that belongs to the IL 6  [ **IL6** ]  family.  Ref: 12855584 Blood< MedlineTA>Blood,
- Oncostatin M  [ **OSM** ]   **OSM**  is a member of the interleukin 6  [ **IL6** ]  cytokine family, which is involved in definitive hematopoiesis, the development of liver, and local inflammation.  Ref: 12831858 Neuroscience, 2003

**Non-interactive relationship** (e.g. studied together, co-existance, homology, etc.)

- Studies with cultured cells and mouse models of disease have ascribed proinflammatory and anti inflammatory functions to oncostatin M  [ **OSM** ]   **OSM**  and the related cytokine, interleukin 6  [ **IL6** ]  IL 6  [ **IL6** ] .  Ref: 12856331 J Interferon Cytokine Res, 2003
- Here, we examined the effect of systemic administration of adenoviral Ad vectors encoding either murine  **OSM**  AdMuOSM or murine IL 6  [ **IL6** ]  AdMuIL 6 in a mouse model of colitis.  Ref: 12856331 J Interferon Cytokine Res, 2003
- after IL 6  [ **IL6** ]  and  **OSM**  stimulation, gp130 dependent pathways STAT3, APR gene expression were completely blocked in the liver of these animals.  Ref: 12891556 Gastroenterology, 2003
- Hepatocyte specific gene expression in BMCs was identified by immunocytochemistry and reverse transcription polymerase chain reaction.RESULTS Bone marrow cell derived hepatocyte like colonies appeared after several days of coculture in medium containing HSLF, oncostatin M  [ **OSM** ]   **OSM**  and hepatocyte growth factor HGF  [ **IL6** ] .  Ref: 12821039 J Hepatol, 2003
- Adult mice were challenged with IL 6  [ **IL6** ] , oncostatin M  [ **OSM** ]   **OSM** , and LPS.  Ref: 12891556 Gastroenterology, 2003
- Transdifferentiation was enhanced by 5 azacytidine treatment, and by HSLF,  **OSM**  and HGF  [ **IL6** ] .  Ref: 12821039 J Hepatol, 2003
